# A systematic review of the menstrual experiences of university students and the impacts on their education: A global perspective

**DOI:** 10.1371/journal.pone.0257333

**Published:** 2021-09-10

**Authors:** Alana K. Munro, Erin C. Hunter, Syeda Z. Hossain, Melanie Keep

**Affiliations:** 1 Sydney School of Health Sciences, Faculty of Medicine and Health, The University of Sydney, Sydney, New South Wales, Australia; 2 Sydney School of Public Health, Faculty of Medicine and Health, The University of Sydney, Sydney, New South Wales, Australia; Jouf University, Kingdom of Saudi Arabia, SAUDI ARABIA

## Abstract

**Background:**

Higher education attainment is linked to improved health and employment outcomes but the impact of university students’ experiences of menstruation on their education is less clear. The objective of this review was to synthesise qualitative and quantitative research on university students’ menstrual experiences and educational impacts.

**Methods:**

Eligible studies were identified through systematic searching across eight peer-reviewed databases, websites for menstrual health organisations, grey literature databases, and reference lists of included studies. Eligible studies must have reported on at least one of the antecedents or components of menstrual experience outlined in the integrated model of menstrual experience in relation to university students or reported on the impact of their menstrual experiences on their education. Study characteristics and findings were extracted, analysed and presented as a narrative synthesis. The quality of evidence was assessed with the Mixed Methods Appraisal Tool. This study is registered on PROSPERO, number CRD42020178470.

**Results:**

Eighty-three studies were eligible for inclusion. Most studies (n = 74; 89%) were quantitative and the highest proportion of studies were conducted in lower-middle-income countries (n = 31; 37%). Self-reported dysmenorrhea, other physical and emotional menstrual-related symptoms, and menstrual stigma contributed to negative menstrual experiences among female students. Very few studies considered the menstrual experiences of non-binary and transgender menstruating students, and culturally diverse students. Dysmenorrhea contributed to university absenteeism, impaired participation and concentration, and declining academic performance. Inadequate sanitation facilities for menstrual management and challenges containing menstruation also negatively impacted education.

**Conclusions:**

Female university students’ experiences of menstruation can negatively impact their education, highlighting the need for program and policy responses at university to improve students’ wellbeing and educational engagement. Further research on the menstrual experiences of gender diverse, migrant and international students is needed as there is insufficient evidence to date.

## Introduction

Menstruation is a natural physiological process experienced by 1.8 billion girls, women, transgender and non-binary persons, globally [1]. There is increased recognition that this experience is not always positive, with a rapidly expanding coalition of academics, non-government organisations, social enterprises, activists and multinational feminine hygiene companies mobilising action to address barriers to menstrual management [2]. A critical impetus has been the mounting research dedicated to understanding adolescent girls’ experiences of menstruation and impacts on their education, particularly in low- and middle-income countries, as a result of the menstrual health and hygiene discourse emanating from the water, sanitation and hygiene (WASH) and education sectors [2, 3]. Recently, there have been calls to explore the experience of menstruation in the workplace [4], with research finding that women’s menstrual experiences impact their labour participation and wellbeing [4, 5].

Higher education attainment is linked to improved personal, family and community health prospects, while benefitting the economy in which individuals work [6, 7]. However, understanding and improving the menstrual experiences of university students has not been a priority of global menstrual health and hygiene initiatives. A greater understanding of how university students’ experiences of menstruation impact their education attainment can inform structural changes (e.g. examination accommodations or design of campus sanitation facilities) so menstruating students are adequately supported to meet their menstrual and learning needs.

Hennegan and colleagues’ [8] integrated model of menstrual experience highlights the multiple components of menstrual experience along with contributing factors and hypothesised impacts. Based on a qualitative meta-synthesis of research into women’s and girls’ experiences of menstruation in low- and middle-income countries, the model illustrates pathways through which distal (sociocultural context and resource limitations) and proximal (social support, behavioural expectations, knowledge, and physical and economic environments) antecedents influence the experience of menstruation which, in turn, impact menstruators’ wellbeing [8].

The objective of this review was to synthesise research on university students’ menstrual experiences globally, and the antecedents to and impact of these experiences on students’ education.

This review sought to extend the current research by: (1) describing the menstrual experiences of students studying in high-income countries, (2) summarising quantitative and qualitative research into students’ experiences of menstruation, predictors of these experiences, and the impact of these experiences on their education, and (3) examining research on the menstrual experiences of students from culturally diverse backgrounds, and non-binary and transgender students.

### Research questions

1. What are university students’ experiences of menstruation?
2. What are the antecedents of university students’ positive and negative experiences of menstruation?
3. How do university students’ menstrual experiences impact their education?
4. How do the menstrual experiences of university students from culturally diverse backgrounds, and non-binary and transgender menstruators differ from students in other groups?

We define students from ‘culturally diverse backgrounds’ as those whose ethnic, racial and/or religious background is different from the dominant ethnicity, race and/or religion of their country of study. This may include migrant or international students. We define non-binary and transgender menstruating students interchangeably with the term ‘gender diverse’.

## Methods

PRISMA guidance was adhered to throughout this review. The review protocol is registered on PROSPERO (registration number CRD42020178470).

### Search strategy and selection criteria

Searches for peer-reviewed studies were undertaken in April 2020 in eight databases: MEDLINE, PsycInfo, ERIC, PubMed, Global Health, Embase, Web of Science and ProQuest. An exemplar search strategy, including how search terms were combined into strings, is enclosed in the supporting materials (S1 Table). Search terms were searched for in all titles, abstracts, and keywords. Reference lists of included full-text articles were manually reviewed to identify additional studies. Websites of menstrual health not-for-profits and organisations (e.g. Menstrual Health Hub, Menstrual Hygiene Day and WaterAid) and grey literature databases (e.g. OpenGrey) were also searched to identify relevant grey literature. An updated search was conducted in December 2020 using additional search terms (e.g. “menstrual health AND university students AND qualitative”) and references lists were reviewed again to screen for any other articles possibly overlooked previously.

#### Participants

All included studies were required to include analysis of menstruating university/college students.

#### Inclusion criteria

Studies had to report on at least one of the antecedents or components of menstrual experience outlined in the integrated model of menstrual experience or reported on the impact of university students’ menstrual experiences on their education. Experiences of withdrawal bleeds from oral contraceptive pills and menstrual disorders (dysmenorrhea, endometriosis, abnormal uterine bleeding, menorrhagia) were also included, but were not exclusively the focus of this review. While disordered menstruation among university students is an important feature of their menstrual experience, this has been captured in systematic reviews elsewhere [9, 10].

#### Exclusion criteria

Reviews and clinical trial studies were excluded. Studies that primarily reported on students’ experiences of menarche, biological characteristics in relation to menstruation (e.g. blood pressure, weight), menstrual-related conditions not listed in the inclusion criteria (e.g. premenstrual syndrome, amenorrhea) or accounts solely given by key informants other than university students were excluded. There was no language exclusion criterion.

### Study selection

Results from the systematic searching were exported into Covidence (https://www.covidence.org/), an online screening and data extraction tool for systematic reviews. Two reviewers (AKM, ZH) independently screened titles and abstracts for eligibility and papers that addressed the menstrual experiences of university students were retained. Full-text articles were then independently reviewed by AKM and ECH for inclusion and discrepancies were resolved through discussion between the two reviewers, and a third reviewer (ZH) if necessary.

### Quality assessment

The quality of eligible studies was appraised using the Mixed Methods Appraisal Tool (MMAT) [11]. Two authors (AKM, MK) co-rated four studies to establish consensus, then independently appraised the quality of the remaining studies with 100% agreement (AKM appraised 80% and MK appraised 20%). Studies were not excluded due to their quality. Findings from high and medium quality studies (indicated as a MMAT score of 40% or higher) were prioritised—extracted data were synthesised together and presented foremost. Findings from low quality studies (MMAT score of 0%-20%) were assessed separately to determine whether they aligned with the conclusions drawn by our synthesis of higher quality studies.

### Data extraction and synthesis

Two authors (AKM, MK) pre-designed a data extraction spreadsheet that enabled data from individual studies to be mapped against each component of menstrual experience according to the integrated model of menstrual experience [8]. Author 1 extracted data on study details (e.g. author, country, study objectives), participant details (e.g. sample size, age, menstruating status, sociodemographics), study design, and key findings into the spreadsheet (S1 Dataset).

Extracted quantitative data were grouped according to each component of menstrual experience (menstrual practices, individual menstrual factors etc.). Within each component, studies with similar research questions were grouped together and AKM categorised findings as similar, conflicting, or one-off. Findings were compared across categories and analytical memos were drafted to summarise the findings. Study designs, survey questions, sample characteristics, country-income classification were also noted as potential explanations for differences in the nature of the findings. These findings were summarised as a narrative synthesis. A meta-analysis was not feasible due to inconsistent study designs and reported outcomes across the included quantitative studies. For qualitative data, direct quotes from research participants and interpretations of the researchers related to any component of the integrated model were extracted. Qualitative data were then synthesised following the same methodology and findings were summarised and presented as text.

## Results

### Study characteristics

**Fig 1:**
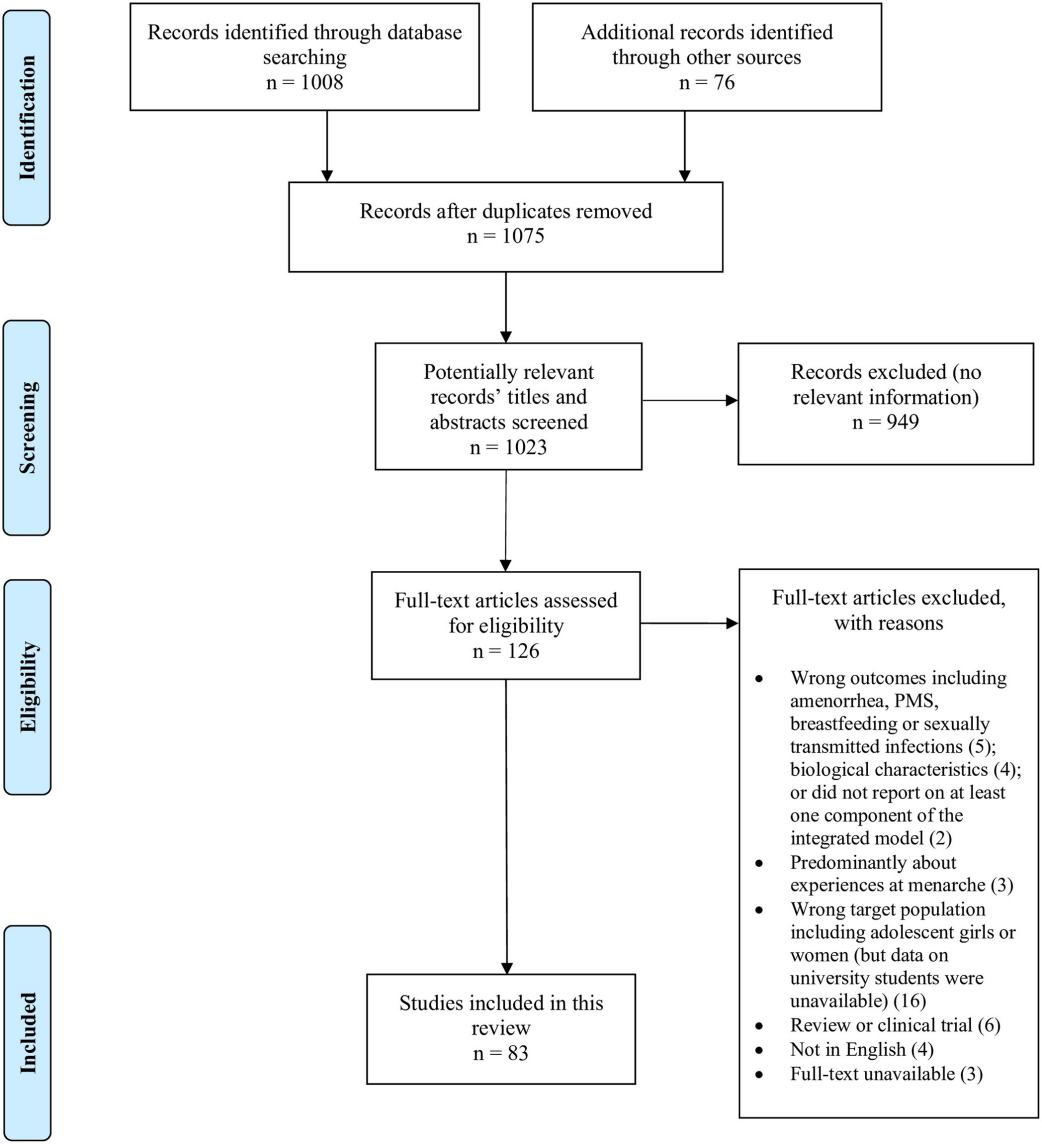
PRISMA flow diagram of the number of records identified, screened, assessed for eligibility and included in this review.

**Fig 2:**
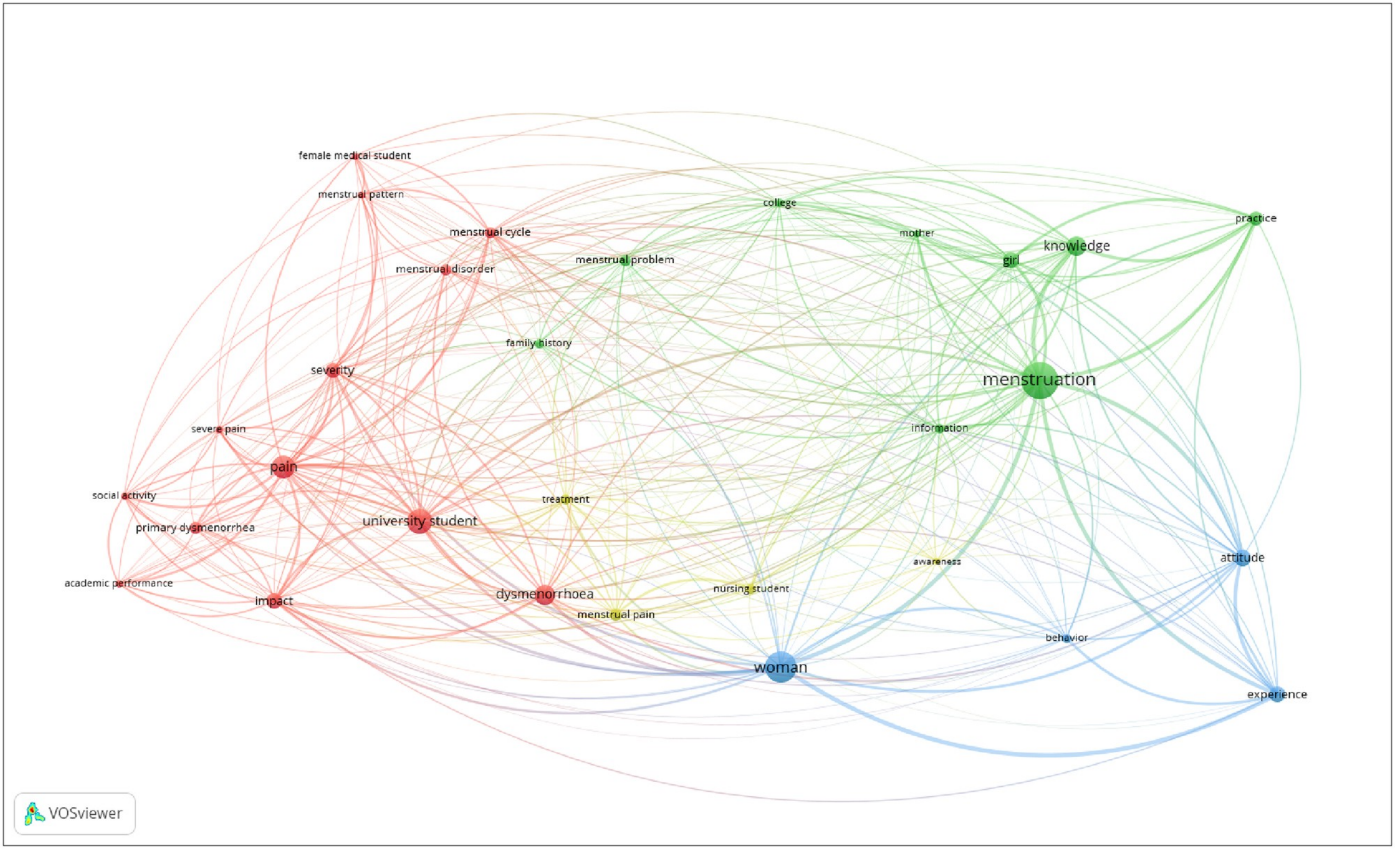
Network map of keyword co-occurrences across the included studies.

The review flowchart is presented in Fig 1. Database searches yielded 1008 records, and reference list searching yielded another 76 potentially eligible studies. After removing duplicates and screening records for eligibility, 83 records were included for analysis. We used the online software tool VOSViewer (https://www.vosviewer.com/) to construct a network map of author-supplied keyword co-occurrence relations across the included studies (Fig 2). The keywords ‘menstruation’, ‘woman’, ‘university student’ and ‘pain’ had the greatest number of co-occurrences.

Table 1 describes the characteristics of the included studies. Most studies were conducted in India (n = 11) and Nigeria (n = 11), followed by Turkey (n = 9) and Saudi Arabia (n = 8). Studies were classified as high-, middle- or low-income using the World Bank Country classification (https://datahelpdesk.worldbank.org/knowledgebase/articles/906519-world-bank-country-and-lending-groups) for the year that the study was conducted or published (if the former was not reported). The greatest proportion of studies were conducted in lower-middle-income countries (n = 31), followed by upper-middle-income countries (n = 25), high-income countries (n = 20) and low-income countries (n = 7). All studies were cross-sectional descriptive studies. Questionnaires were the most common data collection method used (n = 71), with nineteen studies using previously published questionnaires or assessment tools.

**Table 1:** Characteristics of included studies in this review.

Variable	Values	Number (%) N = 83
**Year of publication**	1990–1999	5 (6.02%)
	2000–2009	16 (19.28%)
	2010–2019	58 (69.88%)
	2020	4 (4.82%)
**Study approach**	Quantitative	74 (89.16%)
	Qualitative	8 (9.64%)
	Mixed methods*	1 (1.2%)
**Study country**	India	11 (13.25%)
	Nigeria	11 (13.25%)
	Turkey	9 (10.84%)
	Saudi Arabia	8 (9.64%)
	Ethiopia	4 (4.82%)
	Ghana	4 (4.82%)
	Iran	4 (4.82%)
	South Africa	4 (4.82%)
	Spain	4 (4.82%)
	United States of America (USA)	4 (4.82%)
	China	3 (3.6%)
	Egypt	3 (3.6%)
	Pakistan	3 (3.6%)
	Malaysia	2 (2.41%)
	Taiwan	2 (2.41%)
	England	1 (1.2%)
	Hong Kong	1 (1.2%)
	Jordan	1 (1.2%)
	Lebanon	1 (1.2%)
	Mexico	1 (1.2%)
	Palestine	1 (1.2%)
	Thailand	1 (1.2%)
**Country-income classification when study was conducted** ^**μ**^	High-income	20 (24.1%)
	Upper-middle-income	25 (30.12%)
	Lower-middle-income	31 (37.35%)
	Low-income	7 (8.43%)
**Method of data collection**	Questionnaire only (self-administered/interview by study staff)	71 (85.54%)
	In-depth interviews only	3 (3.61%)
	Focus groups only	3 (3.61%)
	Questionnaire and menstrual diary	2 (2.41%)
	Menstrual diary only	1 (1.2%)
	In-depth interviews and focus groups	1 (1.2%)
	Questionnaire and in-depth interview	1 (1.2%)
	Content (text) analysis	1 (1.2%)
**Time period of study**	1980–1989	2 (2.41%)
	1990–1999	1 (1.2%)
	2000–2009∞	11 (13.25%)
	2010–2019	40 (48.2%)
	2020	2 (2.41%)
	Unreported	27 (32.5%)
**Sample size (range)** €		8–2640

*One study had a mixed design (quantitative [questionnaire] and qualitative [in-depth-interview]).

^∞^Includes two studies in which data were collected over 2009 and 2010.

^€^One study did not disclose sample size.

For this review, we considered Palestine a country, but with West Bank and Gaza country-income classification.

Two studies recruited adolescent high school students alongside university students. Only data from university students were included in this review.

### MMAT appraisal

Quality assessment scores for included studies are available in Table 2. Methodological quality ranged from 0 to 100%. More than half the studies (n = 46, 55.42%) were high quality (MMAT score ≥ 80%), 33 studies (36.76%) were medium quality (MMAT score between 40%-60%), and the remaining four studies (4.82%) were of low quality (MMAT score ≤ 20%). One study received a MMAT score of 0% because it did not report details of the sampling strategy, measurements used to answer the research question, statistical tests and risk of non-response bias, and the sample was not representative of the target population [12]. Findings from lower-quality studies did not differ from findings of higher-quality studies so are reported together.

**Table 2:** Summary of included studies, component(s) of menstrual experience assessed, and quality assessment scores and ratings.

**Quantitative studies**						
**Author, year, country**	**World Bank income group**	**Data collection tool(s)/method(s)**	**Sample**	**Component(s) of menstrual experience assessed**	**MMAT score (%)**	**Quality rating (low, medium, high)**
Abdel-Salam et al. (2018) Saudi Arabia	High	Anonymous self-administered questionnaire	n = 366 age: 18–28	Individual menstrual factors, educational impacts	80	High
Al-Dabal et al. (2014) Saudi Arabia	High	Anonymous self-administered questionnaire	n = 924 age: 18–33, mean = 20.6 (SD = 1.9)	Individual menstrual factors, educational impacts	100	High
Alharbi et al (2018) Saudi Arabia	High	Questionnaire	n = 500 age: mean = 21.1 (SD = 7.8)	Knowledge, experiences of shame/distress	80	High
Alonso and Coe (2001) United States	High	Questionnaire, weekly menstrual symptoms log (diary)	n = 184 age: N/A	Social support, individual menstrual factors	40	Medium
Alsaleem (2018) Saudi Arabia	High	Anonymous self-administered questionnaire	n = 197 age: 18–23, mean = 19.1 (SD = 0.95)	Social support, individual menstrual factors	60	Medium
Anastasakis et al. (2008) England	High	Self-administered questionnaire	n = 767 age: 18–39, median = 22	Individual menstrual factors, educational impacts	40	Medium
Chia et al. (2013) Hong Kong	High	Questionnaire	n = 240 age: mean = 20.1 (SD = 1.4)	Individual menstrual factors, educational impacts	100	High
Chiou & Wang (2008) Taiwan	High	Anonymous self-administered questionnaire	n = 760 age: mean = 16.7	Individual menstrual factors, educational impacts	80	High
Fernandez Martinez et al. (2018) Spain	High	Anonymous self-administered questionnaire	n = 258 age: 18–45, mean = 20.63 (SD = 3.32)	Individual menstrual factors	100	High
Harlow and Park (1996) United States	High	Self-administered questionnaire and menstrual diary	n = 165 age: 17–19	Individual menstrual factors, educational impacts	100	High
Huang and Huang (2020) Taiwan	High	Anonymous self-administered questionnaire	n = 1245 age: 18+	Practices, perceptions of menstrual practices, individual menstrual factors	100	High
Ibrahim et al. (2015) Saudi Arabia	High	Anonymous self-administered questionnaire	n = 435 age: mean = 21.4 (SD = 1.4)	Individual menstrual factors, educational impacts	100	High
Ismaile et al. (2016) Saudi Arabia	High	Questionnaire	n = 100 age: 18–25, mean = 21.81	Individual menstrual factors, educational impacts	40	Medium
Karout (2015) Saudi Arabia	High	Anonymous self-administered questionnaire	n = 400 age: 18–23 (86.8%)	Knowledge	80	High
McPherson and Korfine (2004) United States	High	Self-administered questionnaire	n = 84 age: 18–24	Experiences of shame/distress, individual menstrual factors	60	Medium
Omar et al. (1998) United States	High	Self-administered anonymous questionnaire	n = 250 age: 17–21, mean = 19	Practices, perceptions of practices	40	Medium
Rafique and Al-Sheikh (2018) Saudi Arabia	High	Anonymous self-administered Questionnaire	n = 738 age: 18–25	Individual menstrual factors	60	Medium
Atkas (2015) Turkey	Upper middle	Interviewer-administered face-to-face questionnaire	n = 200 age: 18–36, mean = 20.85 (SD = 2.15)	Individual menstrual factors, educational impacts	100	High
Ansong et al. (2019) China	Upper middle	Self-administered questionnaire	n = 409 age: 17–35, mean = 21.4	Individual menstrual factors	80	High
Baghianimoghadam et al. (2012) Iran	Upper middle	Self-administered questionnaire	n = 300 age: 18–35, mean = 21 (SD = 4.3)	Individual menstrual factors, educational impacts	40	Medium
Cakir et al (2007) Turkey	Upper middle	Anonymous self-administered questionnaire	n = 391 age: 16–27, mean = 20.7 (SD = 1.8)	Individual menstrual factors, educational impacts	100	High
Chen et al (2019) China	Upper middle	Self-administered questionnaire	n = 1231 age: 16–23, mean = 19.21 (SD = 1.026)	Social support, individual menstrual factors	100	High
Cronje and Kritzinger (1991) South Africa	Upper middle	Self-administered questionnaire	n = 102 age: 19–21	Social support, behavioural expectations, practices experiences of shame/distress, individual menstrual factors	40	Medium
Ghiasi (2019) Iran	Upper middle	Questionnaire	n = 282 age: mean = 21.8 (SD = 2.2)	Experiences of shame/distress, individual menstrual factors	100	High
Htut et al. (1996) Malaysia	Upper middle	Self-administered questionnaire	n = 123 age: 20–24	Individual menstrual factors, educational impacts	60	Medium
Kabbara et al. (2014) Lebanon	Upper middle	Self-administered questionnaire	n = 452 age: 18–25	Individual menstrual factors	20	Low
Midilli et al. (2015) Turkey	Upper middle	Self-administered questionnaire	n = 488 age: 18–31, mean = 20.35 (SD = 1.87)	Individual menstrual factors	100	High
Mukattash et al. (2013) Jordan	Upper middle	Interviewer-administered face-to-face questionnaire	n = 2000 age: 15–35, mean = 20.86	Individual menstrual factors	40	Medium
Orhan et al. (2018) Turkey	Upper middle	Anonymous self-administered questionnaire	n = 471 age: 18–25	Individual menstrual factors, educational impacts	100	High
Ortiz (2010) Mexico	Upper middle	Anonymous self-administered questionnaire	n = 1539 age: 17–35, mean = 20.4 (SD = 2)	Individual menstrual factors, educational impacts	60	Medium
Ozerdogan et al. (2009) Turkey	Upper middle	Self-administered questionnaire	n = 800 age: 17–32, mean = 21.47 (SD = 2.02)	Individual menstrual factors	100	High
Padmanabhanunni and Fennie (2017) South Africa	Upper middle	Self-administered questionnaire	n = 255 age: 18 and above	Experiences of shame/distress, educational impacts	60	Medium
Polat et al. (2008) Turkey	Upper middle	Anonymous self-administered questionnaire	n = 1266 age: 17–35, mean = 21.02 (SD = 2.13)	Individual menstrual factors, educational impacts	60	Medium
Potur et al. (2014) Turkey	Upper middle	Self-administered questionnaire	n = 1515 age: 17–34, mean = 20.74 (SD = 2.11)	Individual menstrual factors, educational impacts	100	High
Seven et al. (2013) Turkey	Upper middle	Self-administered questionnaire	n = 371 age: 18–23, mean = 20.31 (SD = 1.1)	Individual menstrual factors, educational impacts	100	High
Tanmahasamut and Chawengsettakul (2012) Thailand	Upper middle	Self-administered questionnaire	n = 552 age: 19 = 24, mean = 21 (1.7)	Individual menstrual factors, educational impacts	80	High
Rakhshaee (2014) Iran	Upper middle	Self-administered questionnaire	n = 600 age: 18–37, mean = 22.3	Individual menstrual factors, educational impacts	80	High
Unsal et al. (2010) Turkey	Upper middle	Anonymous self-administered questionnaire	n = 623 age: 17–30, mean = 20.8 (SD = 1.8)	Individual menstrual factors	100	High
Zukri et al. (2009) Malaysia	Upper middle	Self-administered questionnaire	n = 235 age: mean = 21.4 (SD = 1.8)	Individual menstrual factors, educational impacts	100	High
Abu Helwa et al. (2018) Palestine	High	Anonymous self-administered questionnaire	n = 956 age: mean = 19.73 (SD = 1.5)	Individual menstrual factors, educational impacts	80	High
Aflaq and Jami (2012) Pakistan	Lower middle	Anonymous self-administered questionnaire	n = 245 age: 16–21, mean = 18 (SD = 1.54)	Practices, experiences of shame/distress	80	High
Amaza et al. (2012) Nigeria	Lower middle	Self-administered questionnaire	n = 169 age: 19–46	Individual menstrual factors	80	High
Ameade and Garti (2016) Ghana	Lower middle	Self-administered questionnaire	n = 293 age: 20–25 (75.4%)	Knowledge, practices	100	High
Ameade et al. (2018) Ghana	Lower middle	Self-administered questionnaire	n = 293 age: 20–25 (75.4%), mean = 23 (SD = 5.07)	Individual menstrual factors, educational impacts	100	High
Balla and Nallapu (2018) India	Lower middle	Self-administered questionnaire	n = 254 age: mean = 19.2 (SD = 0.92)	Knowledge, social support, behavioural expectations Physical environment, practices, perception of physical environment, experiences of shame/distress confidence, containment, individual menstrual factors, educational impacts	60	Medium
Charu et al. (2012) India	Lower middle	Self-administered questionnaire	n = 560 age: 17–24, mean = 20.57 (SD = 1.208)	Individual menstrual factors, educational impacts	40	Medium
Ekpenyong et al. (2011) Nigeria	Lower middle	Self-administered questionnaire	n = 393 age: mean = 22.3	Individual menstrual factors, educational impacts	80	High
El-shazly et al. (1990) Egypt	Lower middle	Questionnaire	n = 513 age: 15–22, mean = 17.34 (SD = 1.32)	Knowledge, behavioural expectations, practices, educational impacts	40	Medium
Esimai and Esan (2010) Nigeria	Lower middle	Self-administered questionnaire	n = 400 age: 20–27, mean = 21.1	Individual menstrual factors, educational impacts	60	Medium
Farotimi et al. (2015) Nigeria	Lower middle	Questionnaire	n = 310 age: 13–25	Knowledge, individual menstrual factors, educational impacts	100	High
Gangwar et al. (2014) India	Lower middle	Questionnaire	n = 101 age: 17–30, mean = 19.86 (SD = 2.27)	Individual menstrual factors, educational impacts	20	Low
Iliyasu et al. (2012) Nigeria	Lower middle	Interviewer-led questionnaire	n = 383 age: 17–29, mean = 22.7 (SD = 2.2)	Practices, individual menstrual factors, educational impacts	100	High
Kamel et al. (2017) Egypt	Lower middle	Self-administered questionnaire	n = 269 age: 18–25, mean = 20.4 (SD = 1.7)	Individual menstrual factors, educational impacts	80	High
Kural et al. (2015) India	Lower middle	Self-administered questionnaire	n = 310 age: 17–25	Individual menstrual factors	60	Medium
Loto et al. (2008) Nigeria	Lower middle	Interviewer-led Questionnaire	n = 409 age: mean = 22.13 (SD = 2.94)	Individual menstrual factors	100	High
Manna et al. (2019) India	Lower middle	Anonymous questionnaire	n = 140 age: 18–26, mean = 20.09 (SD = 1.81)	Knowledge, behavioural expectations, physical environment, economic environment, perceptions of environments, perceptions of practices, individual menstrual factors, educational impacts	60	Medium
Mohapatra and Roy (2016) India	Lower middle	Anonymous self-administered questionnaire	n = 441 age: not disclosed, appears approx 18–26	Knowledge, social support, behavioural expectations, practices, perceptions of environments, experiences of shame/distress, confidence and containment, educational impacts	40	Medium
Okoro et al. (2012) Nigeria	Lower middle	Interviewer-led questionnaire	n = 289 age: 18–35, mean = 22.5 (SD = 3.12)	Individual menstrual factors	40	Medium
Okusanya et al. (2009) Nigeria	Lower middle	Self-administered questionnaire	n = 160 age: mean = 22.7 (SD = 2.8)	Individual menstrual factors	40	Medium
Osman and El-Houfey (2016) Egypt	Lower middle	Self-administered questionnaire	n = 188 age: 18–21, mean = 20 (SD = 1.3)	Individual menstrual factors, educational impacts	80	High
Parveen et al. (2009) Pakistan	Lower middle	Self-administered questionnaire	n = 197 age: 18–25	Practices, individual menstrual factors, educational impacts	60	Medium
Sharma et al. (2008) India	Lower middle	Interviewer-led semi-structured questionnaire	n = 100 age: 17–23	Individual menstrual factors, educational impacts	60	Medium
Sharma et al. (2013) India	Lower middle	Anonymous self-administered questionnaire	n = 176 age: 18–26	Behavioural expectations, practices, individual menstrual factors	0	Low
Singh et al. (2008) India	Lower middle	Anonymous self-administered questionnaire	n = 107 age: 17–25, mean = 21 (SD = 2.74)	Individual menstrual factors, educational impacts	60	Medium
Singh et al. (2018) India	Lower middle	Anonymous self-administered questionnaire	sample size not disclosed age: mean = 19.95 (SD = 1.41)	Knowledge, social support, behavioural expectations, physical environment (WASH), educational impacts	20	Low
Yadav and Taneja (2019) India	Lower middle	Self-administered questionnaire	n = 200 age: 17–24, mean = 19.45 (SD = 2.5)	Behavioural expectations, individual menstrual factors, educational impacts	80	High
Yasir et al. (2014) Pakistan	Lower middle	Questionnaire	n = 200 age: 18–25, mean = 21.01 (SD = 1.54)	Individual menstrual factors, educational impacts	60	Medium
Zhou et al. (2010) China	Lower middle	Menstrual diary	n = 2640 age: 16–26, mean = 20.3 (SD = 1.3)	Individual menstrual factors	60	Medium
Adeyemi and Adekanie (2006) Nigeria	Low	Self-administered questionnaire	n = 226 age: 20–35, mean = 25.7 (SD = 2)	Individual menstrual factors	40	Medium
Gebeyehu et al. (2017) Ethiopia	Low	Interviewer-led face-to-face questionnaire	n = 389 age: mean = 21	Individual menstrual factors, educational impacts	80	High
Hailemeskel et al. (2016) Ethiopia	Low	Self-administered questionnaire	n = 440 age: mean = 20.7 (SD = 1.36)	Individual menstrual factors, educational impacts	80	High
Moronkola and Uzuegbu (2006) Nigeria	Low	Self-administered questionnaire	n = 120 age: 21–25 years (73%)	Practices, experiences of shame/distress, individual menstrual factors	60	Medium
Yesuf et al. (2018) Ethiopia	Low	Self-administered questionnaire	n = 242 age: mean = 20.5 (SD = 1.16)	Individual menstrual factors, educational impacts	60	Medium
Zegeye et al. (2014) Ethiopia	Low	Anonymous self-administered questionnaire	n = 470 age: 17–24, mean = 20.4 (SD = 1.2)	Individual menstrual factors	100	High
**Mixed-method studies**						
**Author, year, country**	**World Bank income group**	**Data collection tool(s)/method(s)**	**Sample**	**Component(s) of menstrual experience assessed**	**MMAT score (%)**	**Quality rating (low, medium, high)**
Titilayo et al. (2009) Nigeria	Low	Self-administered questionnaire and in-depth interviews	n = 400 (quantitative), n = 37 (qualitative) Age: <19–34 (quantitative), 18–31, mean = 22 (qualitative)	Social support, economic environment, experiences of shame/distress, confidence and containment, Individual menstrual factors, educational impacts	80	High
**Qualitative studies**						
**Author, year, country**	**World Bank income group**	**Data collection tool(s)/method(s)**	**Sample**	**Component(s) of menstrual experience assessed**	**MMAT score (%)**	**Quality rating (low, medium, high)**
Fernández-Martínez et al. (2020a) Spain	High	Focus groups	n = 33 age: mean = 22.72 (SD = 3.46)	Social support, behavioural expectations, experiences of shame/distress, containment, individual menstrual factors	100	High
Fernández-Martínez et al. (2020b) Spain	High	Focus groups	n = 33 age: mean = 22.72	Experiences of shame/distress, individual menstrual factors, educational impacts	100	High
Ramos-Pichardo et al. (2020) Spain	High	Content (text) analysis	n = 202 age: mean = 21.1 (SD = 2.4)	Individual menstrual factors	100	High
Hosseini and Sadat (2018) Iran	Upper middle	Individual in-depth interviews	n = 8 age: 21–25	Social support, behavioural expectations, experiences of shame/distress, confidence and containment, individual menstrual factors	80	High
Ismail et al. (2016) South Africa	Upper middle	Focus groups	n = 16 age: 18–23	Experiences of shame/distress, containment	40	Medium
Padmanabhanunni et al. (2018) South Africa	Upper middle	Individual in-depth interviews, focus groups	n = 20 age: 20–27, mean = 23	Behavioural expectations, experiences of shame/distress	100	High
Aziato et al. (2014) Ghana	Lower middle	Individual in-depth interviews	n = 8 (excludes high school students) age: 22–38	Knowledge, social support, experiences of shame/distress, individual menstrual factors, educational impacts	100	High
Aziato et al. (2015) Ghana	Lower middle	Individual in-depth interviews	n = 8 (excludes high school students) age: 22–38	Social support, individual menstrual factors, educational impacts	100	High

### Participant characteristics

Participants were university students aged between 15–46 years, although one study did not report participants’ age. Two studies conducted in Saudi Arabia did not report participants’ sex, however, all remaining participants in this review were female. Sample sizes varied from 8 to 2640 participants, with a total greater than 36547. Only ten studies recorded participants’ ethnicity or race, and 18 studies collected data on students’ religion. Only one study investigated the experiences of international students.

### 1. What are university students’ experiences of menstruation?

#### Practices

Of the 83 included studies, 15.7% (n = 13) explored menstrual practices [12–24]. All studies were quantitative, but the way in which these practices were defined and assessed varied substantially across the studies. Type of menstrual material used was the most commonly assessed practice (n = 12) [12–19, 21–24], followed by material change frequency (n = 6) [12–14, 16, 18, 19], bathing and showering (n = 4) [13, 16, 19, 23], genital cleaning (n = 4) [12, 13, 19, 20], handwashing (n = 2) [12, 20], cleaning pubic hair (n = 2) [12, 20], changing menstrual materials at night and at university (n = 1) [14], drying practices for reusable materials (n = 1) [19], and changing undergarments (n = 1) [20].

Two studies explored menstrual practices in high-income countries and only assessed menstrual absorbent type used; the remaining studies were from low- and middle-income countries. Irrespective of country-income level, commercially available pads were the most common product used by university students to manage menses except for students studying in the USA where 81% of students used tampons alone or in combination with pads [22]. Only one study, conducted in Taiwan, assessed menstrual cup usage and found 4.8% of students reported use [17]. When asked how many times they changed their menstrual material, the greatest proportion of students reported changing it at least twice a day [13, 14, 18]. When asked how many materials they used a day, students reported a maximum of three [19] or four pads a day [12]. In an Egyptian study, a minority of university students used up to 8 pads a day [16]. In two studies, undertaken in Eastern India and Ghana, bins were the most popular disposal method for used menstrual materials among students [13, 19]. However, in Southeast India, 74% of students disposed of their pad in a toilet [14]. The included studies did not explore the reasons for different disposal practices.

#### Perceptions

Two studies in India explored students’ perceptions of the suitability of their physical environment for managing menstruation [14, 20], two studies from high-income countries explored menstrual product preferences [17, 22], and another study from India considered both [19]. All studies used cross-sectional surveys with limited opportunities for students to share the extent to which they perceived facilities met their needs. No studies in high-income countries explored students’ perceptions of their environments.

Students’ perceptions of their environments in relation to menstrual experiences were captured through questions about the challenges they faced in executing menstrual practices (n = 2) [14, 19] or reasons for not attending college (n = 1) [20]. Students from India perceived that their university facilities lacked the privacy, disposal systems and water supply necessary to manage menstruation [14, 19, 20].

Students’ perceptions of practices were captured through survey items about advantages and disadvantages of menstrual materials [19], intention to use types of menstrual materials [17], and motivations driving continued use of menstrual materials [22]. Students prioritised comfort over price when choosing menstrual materials, regardless of whether they lived in lower-middle- or high-income countries [19, 22]. In the USA, continued use of a menstrual material type was attributed to comfort and convenience—only 4% of students were influenced by price [22]. Nearly all students in a questionnaire study in India indicated that pads were the ideal material to use during menstruation because they were comfortable, although nearly half of them considered pads expensive [19].

#### Experiences of shame or distress

Sixteen studies explored students’ feelings towards menstruation [14, 15, 20, 21, 24–29]. Of them, nine studies were quantitative [14, 15, 20, 21, 24, 26, 27, 30, 31], six studies were qualitative [25, 28, 32–35], and one employed mixed methods [29]. Overall, most students exhibited negative attitudes toward menstruation irrespective of the income-level of their study country. Quantitative studies associated negative attitudes with poor menstrual knowledge [14], use of home-made pads [24], or fear of leaking/staining menstrual blood on clothing [20] or exposing their menstrual status [15]. A study of students in the USA associated positive menstrual experiences with greater menstrual knowledge, positive health behaviours and good body image [31].

However, there were inconsistencies in how menstrual attitudes were measured across studies. Four studies used standardised questionnaires such as the Menstrual Attitude Questionnaire [26, 27, 31] and the Attitude Towards Menstruation Scale [24]. The remaining studies used survey items on students’ acceptance of menstruation [15, 21], feelings of shame [14], and psychological reactions to upcoming menses [14] or did not report their survey items [30].

In in-depth interviews and focus groups, students discussed their anguish to conceal their menstruation. Students reported menstruation was dirty, evoking considerable distress to keep their menstrual status a secret, especially from men who would find them less desirable if they knew they were menstruating [34, 35]. Students lamented the distress caused by menstrual pain, predominantly dysmenorrhea [25, 28, 29, 33]. Severe menstrual pain was related to depression, regret for being female, a desire to not menstruate, and suicidal ideation to escape their perceived suffering [25]. Spanish students who experienced dysmenorrhea or had female family members that referred to menstruation as a ‘time of sickness’ also constructed menstruation as a ‘disturbance or illness’ or a ‘living hell’ [33]. These students felt a lack of control over their lives as menstrual pain disrupted their daily activities [32].

However, students in upper-middle income countries (South Africa and Iran) also expressed positive sentiments toward menstruation as a confirmation of their womanhood and fertility [15, 28, 34, 35]. In qualitative studies, some students accepted menstruation and menstrual pain as an inevitable part of life, or a blessing, to reduce distress:

*All misfortunes are for women; period*, *giving birth*, *menopause*, *breast and uterus cancer*. *But all these have given women greatness… women tolerate their pain more than men*. *Oh*, *I think a man cannot even endure a moment of period on the first day*, *let alone delivery* [28].

#### Containment and confidence

Five studies (one mixed-method, three qualitative and one quantitative) reported on confidence to contain menstruation. Three of these studies were conducted in middle-income countries and the remaining two studies were conducted in a low- and high-income country, respectively [20, 28, 29, 33, 34]. In in-depth interviews and focus groups with university students, participants expressed feeling fearful of leaking menstrual blood on clothes if unprepared, particularly if experiencing irregular and unpredictable cycles [28, 29, 33]. This diminished their perceived agency to leave their house or interact with others while attending university. In focus groups with students in South Africa, some students stated that they blamed themselves or were criticised by others for failing to contain their menses: *“…everyone is going to know… and everyone will be like she didn’t protect herself and stupid for her because she wore white”* [34]. Spanish university students also discussed the pressure to conceal menstrual symptoms, such as irritability or emotional sensitivity [33]. They perceived this could also draw attention to their menstrual status as these changes normally accompanied their menstrual period [33].

Quantitative studies did not define menstrual confidence or containment. However, in a study in India, 80% of university students indicated that shame and fear of staining their clothes with menstrual blood caused absenteeism [20]. In another questionnaire study with Indian university students, half of the sample reported an inability to cope with menstruation and wished they could disappear when menstruating [14]. The authors did not report whether diminished confidence resulted from containment failures or was linked to other components of the menstrual experience (e.g. a lack of menstrual materials or facilities to manage menstruation) [14].

#### Individual menstrual factors

Across included studies, students’ menstrual characteristics was the most frequently assessed component of the menstrual experience. Seventy-two studies (86.7%) explored menstrual symptoms and the experience of adverse symptoms or disorders was common, regardless of country-income level [12, 14, 15, 17–19, 21, 23, 25, 26, 28–33, 36–90].

Fifty-eight studies reported on students’ experience of menstrual pain, which was generally defined as pelvic pain, abdominal cramping or dysmenorrhea. Fifty-three of these studies employed quantitative designs. The reported prevalence of menstrual pain ranged between 16.38%-90.4% (median = 83.8) [44, 65]. Most students perceived their level of pain as moderate (compared to mild or severe) but thresholds for ‘mild’, ‘moderate’ and ‘severe’ pain were inconsistent. Students reported their pain intensity via a visual analogue scale or numerical scale [15, 18, 37, 39–41, 47, 48, 50, 51, 53–55, 57, 58, 60, 61, 63, 64, 66–68, 70, 77, 82, 84], the Multidimensional Scoring System for Dysmenorrhea [46, 65, 71], questionnaire items on pain severity or the level of interference pain caused to life and work [36, 59, 73, 76, 88], or a mixture of these approaches [23, 72, 80, 90]. Nine studies did not specify their measurement details [14, 30, 31, 42, 43, 52, 78, 79, 86]. For quantitative studies on dysmenorrhea, 21 studies assessed primary dysmenorrhea [37, 41, 47, 48, 50, 51, 55, 57, 58, 63–65, 67–70, 75–78, 80, 83], two studies assessed both primary and secondary dysmenorrhea [23, 62] and 34 studies did not report which type was investigated.

Qualitative research explored students’ experiences with pain. In low- and middle-income countries, students described their pain as excruciating whilst also likening it to a punishment for sinning or a victim of an attack they needed to escape: *“I feel like someone is stabbing me with a knife”* [25]. Spanish students similarly described how pain was debilitating and exhausting, however did not reference it as victimising or punitive.

Common co-occurring physical symptoms included aches and pains in the back, leg, bladder and joints, as well as headaches, weakness, appetite changes, bowel disturbances, skin changes, edema, insomnia and fatigue [15, 18, 25, 71, 83, 89, 90]. Emotional disturbances were equally prevalent; irritability, anger, depression, and nervousness were commonly associated symptoms of primary dysmenorrhea among students [36, 37, 39, 46, 47, 54, 62, 78, 83, 89, 90]. Irregular periods were also a salient part of students’ menstrual experiences in both high- and middle-income countries, and self-reported prevalence ranged from 2.2%-74.1% (median = 27%) [15, 37, 38, 41, 44, 46, 49, 54, 56, 69, 73, 75, 79, 84]. Subjective menorrhagia (heavy bleeding), oligomenorrhea (light bleeding) and abnormal menstrual flow (<2 days, >7 days) were less frequently reported among participants in low- and middle-income countries [29, 44, 46, 56, 69, 79, 84].

Fifty-two studies (62.65%) assessed how students coped with troubling symptoms and strategies varied. Analgesics were a key coping strategy to relieve menstrual pain for students irrespective of country-income level [15, 18, 30, 38, 41, 52, 60, 79, 82–85, 89, 90] and greater severity of menstrual pain was related to increased use [38, 52, 60, 63, 80]. Non-pharmacological strategies were also recorded and differed for students within high-income countries. In Saudi Arabia and Taiwan, students were more likely to use herbal medicines, apply heat or rest than take analgesics for pain management [36, 41, 55, 78], but most students studying in Hong Kong used a combination of anti-inflammatory drugs, herbal medicines and dietary supplements [48]. Across low- and middle-income countries, quantitative studies revealed that rest, heat application, consumption of hot beverages, positive self-talk, distraction, herbal remedies, showering and religious prayers were popular coping strategies [39, 42, 46, 52, 57, 60, 79, 82, 84, 86]. Overall, physical activity to relieve pain was less common among students [38, 52, 60, 84, 90].

Only three studies, conducted in low- and middle-income countries, provided insight into why students may adopt non-pharmacological management strategies as opposed to pain killers [29, 45, 84]. In the questionnaire study, students indicated they were primarily concerned of possible side effects of pain killers. In in-depth interviews, students discussed that a fear of addiction, previous adverse reactions and overall ineffectiveness in relieving pain discouraged use [29, 45, 84].

Independent of whether students studied in low-, middle- and high-income countries, most did not consult a health professional for their menstrual-related complaints [30, 41, 43, 52, 54, 61, 62, 64, 78, 79, 83, 84, 89–92]. Among Spanish students, there was an overwhelming belief that pain was normal and something that most women experience, and thus medical advice was unnecessary [92]. However, they also cited that they did not have the time to see a doctor; thought their doctor would trivialize the pain or prescribe them analgesics or birth control pills instead; they preferred to endure the pain, or decided to self-manage it through medication or non-pharmacological approaches. South African students who sought medical advice felt their doctor lacked understanding of their concerns [15]. Students in England were more likely to visit a doctor for subjective menorrhagia compared to normal or light periods, and were less likely to see a doctor for dysmenorrhea even if pain was severe [43].

### 2. What are the antecedents of university students’ positive and negative experiences of menstruation?

#### Knowledge

Sufficient knowledge of menstruation was positively associated with good menstrual hygiene and positive menstrual attitudes in students across low-, middle- and high-income countries [13, 14, 20, 31]. However, the studies measured menstrual knowledge differently and used different sets of menstrual practices to determine ‘menstrual hygiene’. Qualitative studies with students in low- and middle-income countries described how deficits in practical menstrual knowledge led to negative menstrual experiences as students tried to interpret and apply inaccurate advice from friends and doctors in managing dysmenorrhea [25, 29]. Students were advised that frequent sex, childbirth, and marriage would decrease menstrual pain [25, 29, 93].

#### Social support

Across low-, middle- and high-income countries, mothers and friends were important sources of support for students coping with distressing menstrual pain and substituted for medical attention [14, 24, 29, 41, 47, 78]. Students indicated in quantitative surveys that their friends provided a buffer against negative menstrual experiences by offering emotional support and advice for managing menstrual pain, completing their daily tasks when pain interfered, and teaching class content they missed when dysmenorrhea inhibited lecture attendance [14, 41, 47]. In in-depth interviews, students described how their friends’ empathy helped them cope with dysmenorrhea: “*Sometimes the way they [friends] talk to me ‘you will be fine’*. *Just a touch and I feel ok; the person understands what I am going through”* [45]. Similarly, only one study (qualitative) with students from a lower-middle income country mentioned how a work colleague would cover shifts when a student’s menstrual pain was too severe to attend work [25].

Irrespective of country-income level, quantitative studies reported that students’ social support was associated with dysmenorrhea prevalence. An Ethiopian study found that students were at greater risk of primary dysmenorrhea if they experienced a previous disruption to their social network (family, friends, or previous relationships) compared to students who did not, although the authors did not report how ‘disruption’ was measured [80]. Similar findings were reported in a study with American students, where disruption was measured as ‘total loss’ of support using the Norbeck Social Support Questionnaire [81].

In qualitative studies, students across low-, middle- and high-income countries reported various levels of support from males. Some students described how husbands and fathers purchased menstrual materials on their behalf or were understanding when they told them about their dysmenorrhea [28, 33, 45]. Yet, others felt isolated and angry with men when they were in pain because men could not empathise [28, 33]. A lack of empathy was a critical factor in shaping students’ interactions with employers and healthcare workers and contributed to negative menstrual experiences. Spanish nursing students hesitated to tell their boss that they could not attend work because of dysmenorrhea [33]. They feared their female colleagues would trivialise their pain if they had not shared a similar pain experience [33]. Similarly, Ghanian students reported doctors and nurses were dismissive of their dysmenorrhea, resulting in anger and overall distrust in health professionals in treating menstrual health:

*When you go and you are in pain*, *they rush to you but as soon as they realize it is dysmenorrhea*, *they relax*. *They say this thing will not kill you so they leave you on the bed and you see them attend to other people* [45].

#### Behavioural expectations

Seven quantitative studies and one qualitative study conducted in low- and middle-income countries (India n = 5, South Africa n = 2), reported that students followed behavioural proscriptions during menstruation [12, 14, 15, 19, 20, 73, 94]. No studies with students from high-income countries explored behavioural proscriptions to enable comparisons. Quantitative studies measured the prevalence of these behaviours differently; five studies asked students to indicate the restrictions they adhered to [12, 14, 19, 20, 94], one study included questions on students’ awareness of ‘taboos’ [73] and one study did not report their measurement [15].

Restrictions affecting university students primarily concerned religious activities including prohibitions from entering places of worship, reading religious texts, attending religious ceremonies, offering prayers or touching holy books [12, 14, 19, 20, 73, 94]. Others practised dietary restrictions [12, 14, 19, 73], refrained from exercise or sports [14, 15, 73, 94], did not enter the kitchen or prepare food [12, 14, 73], were secluded from friends or family [14], or were required to sleep separately [19, 73], In focus groups with students in South Africa, they expressed mixed feelings toward these restrictions [35]. Whilst some viewed restrictions as respite from chores, others found it unfair and restrictive, but feared repercussions from family and religious leaders if they did not follow them. A questionnaire study in India found that students were less likely to adhere to these restrictions when they lived away from home [12].

Irrespective of country-income level, students’ experiences of shame led to self-imposed expectations of their own behaviour during menstruation. Both qualitative and quantitative research highlight that students internalised menstrual stigma concerning visible menstrual blood which negatively impacted their confidence to attend university or go out in public [16, 20, 33, 35, 94]. In in-depth interviews and focus groups, university students discussed how they avoided or regulated their behaviour in the presence of males when menstruating to keep their menstrual status a secret [28, 33, 35]. Results from a quantitative study in South Africa suggests this affects help-seeking behaviour; university students preferred to consult with female than male doctors regarding menstrual symptoms (69.9% vs 1.9%) [15].

#### Physical and economic environment

In lower-middle income countries, campus facilities were characterised as unhygienic, lacking continuous water supply or dustbins, and without privacy [19, 20, 94]. This influenced how often students could change their menstrual product, their disposal choices and ability to conceal menstruation, which led to shameful experiences [19, 20, 94]. The paucity of studies in high-income countries prevented comparisons between country-income groups.

In lower-middle income countries, unaffordability of menstrual materials contributed to negative menstrual experiences. During in-depth interviews with students in Nigeria, one student with menorrhagia stated that the high costs of pads exacerbated feelings of distress during menstruation: *“Even the extra spending on the pad every month is enough as a discomfort in my own case*.*”* [29]. When only home-made pads were available to students in Pakistan, they were more likely to experience emotional disturbances and experience interruptions to daily routines [24].

### 3. How do university students’ menstrual experiences impact their education?

Educational consequences of the menstrual experience included absenteeism (n = 43, 51.8%) [14, 16, 18–20, 23, 27, 29, 32, 36, 37, 39, 40, 42, 43, 46, 48, 51–55, 57, 63–65, 67, 68, 70, 71, 73, 74, 77–80, 82–84, 86, 90, 94], participation and concentration in class (n = 17, 20.5%) [25, 29, 30, 32, 36, 42, 46, 48, 52, 63, 70, 74, 78, 80, 84, 89, 90], or academic performance (n = 14, 12.9%) [27, 30, 32, 37, 45, 46, 49, 52, 57, 70, 79, 80, 84, 87]. Quantitative studies assessed these impacts through students’ self-reports, however only seven studies reported details of their measurements [18, 48, 53, 55, 63, 67, 84]. These included questionnaire items on: students’ absenteeism due to dysmenorrhea in the last six months [48], the ‘level of interference’ menstruation had on attending lectures and completing assignments [18], history of absence from class due to menstrual pain [55], number of missed days and examinations due to menstrual pain in the last 12 months [67], number of days of missed university due to pain in one month [63], whether dysmenorrhea caused ‘course absenteeism’, ‘lack of concentration in class’ or ‘decrease in course grade’ [84], or how students rated their academic performance or ability to concentrate in class during painful menstruation [63, 67]. One study, conducted in the USA, asked students to record their menstrual cycle and days of absence in a menstrual diary over 12 months to determine menstrual-related absenteeism [53]. No studies compared students’ responses with extant data (i.e. official academic and attendance records) to validate their assertions.

In quantitative studies that collected data on full and partial day (i.e. individual lectures) absenteeism, students reported missing both full and partial days of university because of menstrual-related symptoms and disorders [51, 71, 78]. Full day absenteeism ranged from 1 to 7 days, but studies did not report whether this was per menstrual period or per academic year [18, 40, 67]. Irrespective of country-income level, students indicated that dysmenorrhea prominently contributed to absenteeism, reduced concentration and engagement within the classroom, and declining academic performance. However, the size of this impact varied across the studies [42, 46, 48, 49, 70, 74]. Dysmenorrhea appeared to present a greater barrier to students’ academic performance and concentration than their attendance [46, 48, 52, 57, 70, 80, 84, 90]. Students with severe pain had significantly greater rates of absenteeism and reduced engagement compared to those with mild, moderate or no pain [46, 63, 65, 70].

Qualitative studies provided an in-depth understanding of how students viewed these impacts. Students expressed frustration at themselves for their study and class concentration limitations [32] or at their university for failing to acknowledge how pain can impact class attendance and achievement [25, 29]. To reduce this impact, students took painkillers [32, 65, 80, 82] or completed university assignments before their next period:

*…if I have an assignment that will be due during my menses… I try to do them before my menstrual period*. *I also learn ahead of time because I know I cannot learn with menstrual pain* [45].

In focus groups, Spanish students with dysmenorrhea described how they would attend their compulsory classes despite being in pain, but otherwise would miss class because the pain was too intense and they could not focus [32].

Aside from pain, two quantitative studies conducted in a high- and a lower-middle-income country found that students with heavy menstrual bleeding absented from lectures or full days at university [14, 43]. Absenteeism was also pronounced in students who perceived their university facilities as unsanitary and lacking water, privacy and dustbins as they could not comfortably manage their menstruation [71]. Fear of leaking or staining clothes with menstrual blood discouraged university attendance as students’ menstruating status became easy to detect, highlighting how experiences of shame and distress, coupled with perceived concealment failures, are detrimental to educational achievement [16, 20, 94]. In contrast, a quantitative study with undergraduate nursing students in India found that 97.7% of students continued to attend university while menstruating [19].

### 4. How do the menstrual experiences of university students from culturally diverse backgrounds, and non-binary and transgender menstruators differ from students in other groups?

The menstrual experiences of culturally diverse and gender diverse students were under-researched and were limited to quantitative studies conducted in low- and middle-income countries.

#### Culturally diverse backgrounds

Two studies (2.4%), conducted in Ghana, explored associations between students’ religion and their menstrual practices or experiences of menstrual disorders [13, 42]. Muslim students demonstrated greater menstrual hygiene compared to Christian students, which was defined as using a pad, changing a used pad at least twice daily, disposing a used pad in a bin, and at least bathing on the first day of menses [13]. Self-reported dysmenorrhea was more prevalent in Ghanian students who followed Christianity compared to Muslim students, however this was not statistically significant [42].

Although five studies captured data on students’ ethnicity [18, 47, 66, 75, 77] only one reported on how menstrual experiences differed across ethnic groups and this was limited to the experience of disordered menstruation [77]. In Malaysia, more students who identified as Indian or Chinese reported experiencing dysmenorrhea compared to Malaysian students, but this difference was not statistically significant [77].

A study of students in South Africa explored the relationship between race and students’ attitudes toward menstruation [27]. While findings were not statistically significant, black and coloured students were more likely to perceive menstruation as debilitating compared to their white counterparts. They were also more likely to deny that menstruation can cause emotional distress and bothersome cramps compared to white students [27].

Only one study, conducted in China, addressed international students’ menstrual characteristics, but did not compare their experiences to local students’ [44]. After arriving in China to study, nearly half of the participants reported changes to their menstruation, including irregular menstruation, abnormal amount of menstrual blood, dysmenorrhea, abnormal menstrual cycle length and abnormal bleeding duration [44].

#### Non-binary and transgender menstruators

Eighty-one studies (97.6%) assessed female students’ experiences of menstruation, and two studies did not report the gender of participants [40, 56]. Therefore, no data were available on the menstrual experiences of non-binary and transgender menstruating students to compare with female students.

## Discussion

Overall, students reported negative experiences of menstruation, attributed mainly to dysmenorrhea or menstrual stigma. Experiences were characterised as distressing and shameful, which diminished their sense of agency and confidence to contain menstruation and posed negative consequences to their educational engagement and psychosocial wellbeing.

Interestingly, this review found that, in qualitative studies, a small cohort of university students with dysmenorrhea in South Africa and Iran found menstruation a positive experience for symbolising their femininity. One potential explanation could be the influence of questions asked and methodologies employed in these studies. From the example questions and descriptions made available in these papers, it appeared that students were asked open-ended questions on their feelings toward, and experiences of, menstruation which enabled the collection of positive and negative responses. In contrast, the research aims of the remaining qualitative studies in Ghana, Nigeria and Spain were to understand how students described the negative impacts or restrictions dysmenorrhea placed on their education and their daily activities. Another explanation could be the high proportion of religious women in these studies. Cohen et al. [95] reports that religious proscriptions operate within a larger purity system that promotes the auspiciousness of women and the notion of menstruation as a sacred power that is viewed positively by women [95]. Therefore, students experiencing painful menstruation could view it as a positive experience and a way of connecting them to their religious faith.

Few studies, especially from high-income countries, focused on students’ perceptions of the extent to which their campus sanitation facilities enabled them to execute their preferred menstrual practices. Depending on students’ practices, students may perceive that campus facilities are not equally accessible to them despite being of the same design and standard. Students using menstrual cups as their preferred menstrual material will require access to clean water to clean and reuse it in privacy [96]. These requirements differ to students using commercial pads or tampons that may have sanitary bins or other preferred disposal options available to them. Further research exploring university students’ perceptions of their physical environment in enabling them to carry out their preferred menstrual practices would ensure that interventions implemented to modify university sanitation and hygiene infrastructure meets their specific menstrual needs.

Restrictions were commonly depicted as coercive and oppressive by women and girls for limiting their social, educational, and economic participation. We found that these proscriptions could also advantage students by exempting them from daily chores. Women from Hindu and Islamic faith who described restrictions as coercive and unacceptable also spoke of how restrictions provided an opportunity to rest, or have a break from chores or religious activities during menstruation [97]. Moreover, we found that most students living away from home stopped following restrictions. A potential explanation is that these students became exposed to different information on menstruation and open communication between friends from other cultures that prompted them to question the restrictions. Burchard, Laurence and Stocks [98] found that female international students in Australia exhibited divided opinions towards premarital sex despite it being taboo in their home country. Some students saw separation from parents as providing greater freedom to engage in sexual activity [98]. Since students move away from family to pursue higher education in many contexts [99], there is merit in investigating the effect of relocation on perceptions of, and adherence to, expected behaviours and how this impacts students’ menstrual experiences.

Consistent with previous research into high school and tertiary students [100], dysmenorrhea had negative consequences to students’ university education regardless of their study country’s income level. From qualitative findings, we learnt that students adopted strategies, such as completing assignments before their period or taking medication, to mitigate the impact of pain on their academic performance and attendance. This indicates students’ resourcefulness and adaptability in the pursuit of their education. It also challenges the deficit narrative which positions menstruators as passive recipients of this recurring bodily process that places limits on their participation and ignores how menstruators are proactively overcoming barriers to improve their experiences. Further research could explore other strategies students employ to mitigate adverse menstrual-related impacts to other areas of their life because this can help identify potential systemic barriers that could be removed to support their psychosocial outcomes.

There was a bias in the literature towards reporting on female university students’ menstrual experiences, but research suggests that the experiences of non-binary and transgender persons are not equal to their cisgender peers. In a study of masculine of center persons and transgender men, 32% and 42% of participants who experienced menstruation found it difficult or very difficult to manage their menstruation at school or work, and in other public places, respectively [101]. Participants felt anxious using male restrooms when menstruating and chose to either avoid using them or take steps to secretly change their menstrual material because they feared transphobic attacks if discovered [101]. Policies and interventions informed by the experiences of cisgender students’ experiences as captured in this review will fail to appropriately equip genderdiverse students to meet their menstrual needs while at university. If the menstrual experiences of this cohort are better understood, practitioners can design university-based menstrual health interventions better suited to their needs.

Similarly, few studies explored the experiences of culturally diverse students. Research with migrant and refugee women in Australia and Canada showed that their constructions of menstruation evolved since residing in their host country; they questioned their motivations for adopting traditional rituals during menstruation and chose to stop following them or do so in an adapted form [102]. University students may also experience challenges or opportunities when navigating their cultural expectations of menstruation that might conflict with the expectations of their classmates. The extent to which this occurs and how this shapes their menstrual experience is unclear, but worth investigating to determine what support culturally diverse students need.

Most studies used cross-sectional surveys to assess university students’ menstrual experiences. This provides a snapshot of how students’ experience menstruation at a given point in time but cannot explain if, how and why these experiences change over time. Qualitative research can provide an in-depth exploration of students’ beliefs and perceptions toward their menstruation that is not afforded through a quantitative paradigm, however only eight studies employed qualitative methodologies. Undertaking both quantitative and qualitative research with university students will provide a holistic insight into their menstrual experiences and impacts on their education.

### Limitations

Despite our comprehensive search strategy, we may have overlooked studies eligible for inclusion in this review due to inconsistent concept definitions and measurements. This review further supports calls for establishing consensus around validated measurement tools that enable improved assessment of core concepts, and we acknowledge the work of Hennegan and colleagues in progressing these priorities [103, 104]. Not all studies presented participants’ demographic data such as socioeconomic status, parental education, and place of residence (urban or rural), which limited our ability to explore the effect of these factors on outcomes and explain contradictory findings. Further, the impact of menstrual experiences on education may be underestimated as students experiencing severe pain or resource deficits may have withdrawn from their education and thus were not captured in the studies included in this review. As with all systematic reviews, there is a risk of publication bias and we have tried to mitigate the effects of this by considering grey literature [105].

## Conclusions

University students generally report negative experiences of menstruation, attributed to menstrual pain, experiences of shame and distress, and difficulties containing menses. These factors adversely affect their education through absenteeism, reduced engagement, and poor academic performance. However, menstruation can be a positive experience for some students, and their ability to adapt to challenges presented by dysmenorrhea demonstrates their resilience and creativity. Our findings emphasise how menstrual health interventions that safeguard students against the negative impacts on their education have a place at university, but further research is necessary to appropriately inform design and implementation of these interventions, particularly with students from culturally diverse backgrounds, and those identifying as non-binary or transgender.

## Supporting information

S1 Dataset Extracted data from included studies mapped to each component of menstrual experience. (XLSX)

S1 Table Indicative search strategy for MEDLINE. (PDF)

S1 Checklist (DOCX)
