# Supplementary material for: A systematic review of the menstrual experiences of university students and the impacts on their education: A global perspective
Source: PLoS One. 2021 Sep 10;16(9):e0257333. doi: 10.1371/journal.pone.0257333 (PMC8432759; doi:10.1371/journal.pone.0257333)
Supplement: S1 Table — (PDF) [file pone.0257333.s002.pdf]

Indicative Search Strategy

Database search terms are displayed for MEDLINE and were adapted accordingly for the additional databases.

**Search 1: Menstruation**	Menstruation/ OR<br>Menstrua*.mp. OR<br>Menstrual Cycle/ OR<br>Menstrual Cycle*.mp. OR<br>Mense*.mp. OR<br>Menarch*.mp. OR<br>Catameni*.mp. OR<br>Menstrual Period*.mp. OR
**Search 2: Experiences**	Hygiene/ OR<br>Hygiene*.mp. OR<br>"health and hygiene*".mp. OR<br>Health Knowledge, Attitudes, Practice/ OR<br>(Health* adj3 (knowledge* or attitude* or practice*)).mp. OR<br><br>AND<br><br>Challeng*.mp. OR<br>impact*.mp. OR<br>Barrier*.mp. OR<br>Opportunit*.mp. OR<br>Effect*.mp. OR<br>Effect*.mp. OR<br>Affect*.mp. OR
**Search 3: University students**	University student*.mp. OR<br>Universities/ OR<br>Universit*.mp. OR<br>College*.mp. OR<br>College student*.mp. OR<br>Tertiary education*.mp. OR<br>Campus student*.mp. OR
**Search 4: Education**	Absenteeism/ OR<br>Absenteeism*.mp. OR<br>Attendance*.mp. OR<br>Drop?out.mp. OR<br>Failure*.mp. OR<br>Status*.mp. OR<br>Achievement/ OR<br>Achievement*.mp. OR<br>Engag*.mp. OR<br>Perform*.mp. OR<br>outcome*.mp. OR
**Final search**	1 AND 2 AND 3 AND 4
